# Performance Problems of Non-Toroidal Shaped Current Transformers

**DOI:** 10.3390/s20113025

**Published:** 2020-05-26

**Authors:** Carlos A. Platero, José Ángel Sánchez-Fernández, Konstantinos N. Gyftakis, Francisco Blázquez, Ricardo Granizo

**Affiliations:** 1 Department of Automatic Control, Electrical and Electronic Engineering and Industrial Informatics, Universidad Politécnica de Madrid, E-28006 Madrid, Spain; francisco.blazquez@upm.es; 2 Department of Hydraulic, Energy and Environmental Engineering, Universidad Politécnica de Madrid, E-28040 Madrid, Spain; joseangel.sanchez@upm.es; 3 School of Engineering, The University of Edinburgh, Edinburgh EH9 3FB, UK; k.n.gyftakis@ieee.org; 4 Department of Automatic Control, Department of Electrical, Electronic, Automatic Engineering and Applied Physics, Universidad Politécnica de Madrid, 28012 Madrid, Spain; ricardo.granizo@upm.es

**Keywords:** instrument current transformer, current sensor, IEC 61869 tests, iron core saturation

## Abstract

Nowadays, non-toroidal shape primary pass-through current transformers are commonly used for large current machines with several cables per phase. As these transformers exhibit no radial symmetry, it is not clear if they can be tested using the indirect test described in the IEC 61869 standard. In order to answer this question, two non-toroidal shaped current transformers of different secondary winding designs have been tested and simulated. One transformer has a uniformly distributed secondary winding and the other has a partially distributed secondary winding. Both transformers have the same nameplate characteristics. Both perform correctly in the indirect test. However, only the transformer with the uniformly distributed secondary winding performs correctly in a direct test. A finite element simulation shows that the iron core of the partially distributed secondary winding transformer was saturated, while the iron core of the uniformly distributed one was not. This result explains their different performance. The main conclusion is that the indirect test is not sensitive enough to cover all cases and therefore under doubtful situations, the transformers should be tested using the direct test.

## 1. Introduction

Safe operation of power systems requires good performance of protective relays. While the continuous growing energy demand causes the expansion of power systems, their fault current levels increase. So, in order to achieve a proper operation of the protective relays, a dependable measure of these currents is needed. This measurement uses current transformers to adapt the high fault currents flowing through their primary to a range that can be managed by protective relays in their secondary winding [1]. In addition, electrical machines monitoring is usually performed by measuring currents. For the above mentioned reasons, these current measurements use current transformers. Examples of these are in transformer differential protection [2], or generators and motors [3]. Moreover, the detection of arc faults in low voltage alternating current systems can be achieved using current transformers [4].

Currents flowing through the secondary windings of current transformers should be directly proportional to the currents flowing through their primary windings. In this way, measuring secondary currents is an effective indirect way of measuring the primary currents. This is the usual way of measuring large currents in power systems and electrical machines. The above mentioned linear performance happens under the condition that the magnetic core of the current transformer is not saturated [5]. However, if the transformer’s iron core is saturated, the estimated values of the primary currents obtained through measuring the secondary currents are lower than the actual ones [6], leading to malfunctions in the protection systems.

There are several methods to compensate the effect of the saturation [1]. Recent developments in this approach include time frequency analysis [7], discrete Fréchet distance algorithm [2], histogram-based methods [8], Kalman filter [9], empirical mode decomposition and Savitzky-Golay filtering [10], morphological gradient and decomposition [11], improved gradient [12], wavelet-based [13] and deep learning approach [14]. One possible cause of saturation is due to the presence of a direct current (DC) component in the primary current. In this case, a specific compensation procedure must be applied [15]. To this aim, a detection of this DC current is needed. This can be made injecting a high frequency to the secondary winding [16]. In addition, it is possible to use sensors specific for DC currents [17]. Another popular option is using DC tolerant current transformers [18].

Another option is to modify the magnetic core in order to make it less prone to saturation, by adding an air gap in the magnetic core [19]. In this case, usual compensation methods do not work, so a specific method should be used instead [20].

Nowadays, there are many non-linear loads and non-conventional generations in power systems. Therefore, the currents and voltages could be distorted in some power system nodes. Up to now, the calibration procedures have taken into consideration only sinusoidal waveforms, however, there is some recent research about the applicability of current transformers in the new scenario [21].

In any case, standards specify accuracy classes of current transformers [22,23,24]. According to IEC 61869 standard [23], two parameters define an accuracy class. The first one is the highest allowed percentage of the composite error at the rated limit primary current. The second one is the Accuracy Limit Factor (ALF), which is the ratio of the rated accuracy limit primary current to the rated primary current.

To test the accuracy of current transformers, the IEC 61869 standard proposes two methods. The first one involves injecting currents of 1, 5, 20, 100, and 120% of rated primary current through the primary winding. A resistance (defined by the standard) is connected to the secondary winding. This is a direct method that has the drawback that it needs the injection of a very high primary current. The other method is an indirect one. It is based on checking the knee point of the iron core magnetic characteristic. The test measures the excitation current when the secondary winding has a voltage source and the primary winding is open. The knee point is reached when a 10% secondary voltage increase corresponds to a 50% increase in the excitation current. According to the IEC 61869, this indirect method may be applied only if the current transformer has a low leakage reactance.

Nowadays, non-toroidal shape primary pass-through current transformers are commonly used for large current machines with several cables per phase. In this type of current transformer, the cables that carry the current feeding the machine pass through the transformer window. The shape of the core is elongated and non-toroidal. This allows the use of several cables per phase in an easy way [25].

This research was motivated by the experience of an utility, which had used a set of non-toroidal current transformers in some switchgears. All of them were successfully tested according to the indirect method defined in IEC 61869 standard [23]. However, concerns were raised when an external fault caused a trip in a differential protection of a power transformer.

In [25], two similar non-toroidal shape current transformers with identical specifications were tested following both indirect and direct methods. Both CT’s presented similar results in the indirect method, which according to the standards would assure the correct operation of both of them. However, when testing them with the direct method, while an actual high current was flowing into the primary winding, one of them operated as expected and the other did not.

In order to explain these results, two non-toroidal shape current transformers have been built using the same magnetic core and two different secondary windings. The performance of both transformers was analyzed through simulations with the finite element method and later they were subjected to experimental testing. In Section 2, the characteristics of the specially manufactured transformers are given. In Section 3, the results of the experimental measurements and the simulations are presented. Section 4 critically discusses the results, and finally, Section 5 summarizes the conclusions of this paper.

## 2. Materials and Methods

**Figure 1:**
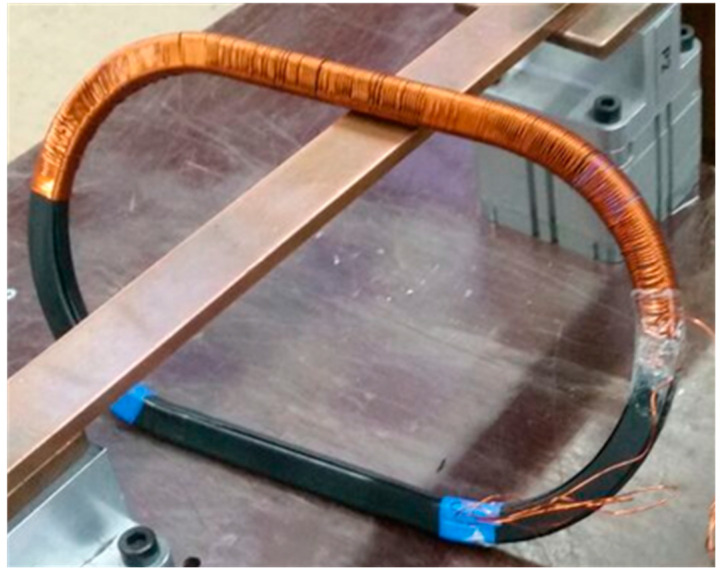
Current transformer core with partially distributed secondary winding.

**Figure 2:**
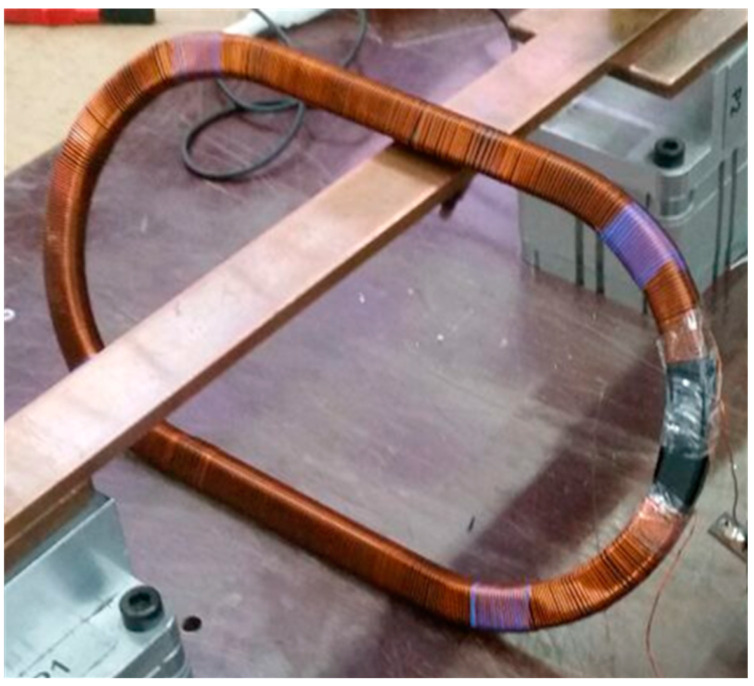
Current transformer core with distributed secondary winding.

As previously shown in [25], two similar current transformers with analogous iron core saturation, may present a different behavior at high current operation. In order to continue towards the same direction, two transformers were built with similar characteristics. The magnetic cores were identical. The difference between the two lies in the fact that one of them has its secondary winding distributed only along some part of the core (Figure 1). On the other hand, the other has its secondary winding distributed along the entire core (Figure 2). Notwithstanding, both transformers have the same electrical characteristics, summarized in Table 1.

The performance of both transformers was tested according to the direct test method described in IEC 61869-2 [23]. So, both transformers were tested under primary currents supply between 500 and 5000 amperes. Their corresponding secondary currents should be between 1 and 10 amperes. The tests were made controlling the primary current and measuring secondary current and voltage. So, the internal resistance of the secondary winding can be calculated.

**Figure 3:**
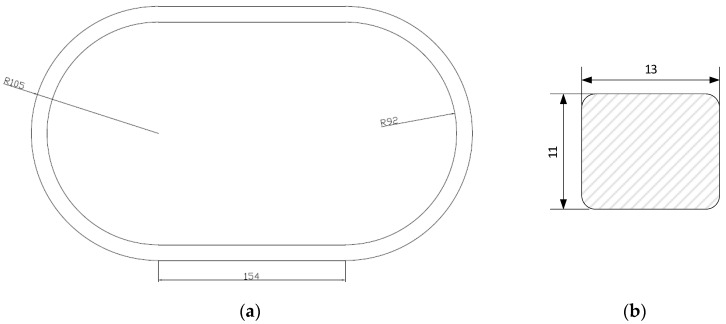
Current transformer (**a**) dimension and (**b**) core cross-section (Lengths in millimeters).

In addition, the performance of these transformers has been simulated using the FEMM software [26]. In order to perform the simulations, the geometrical and magnetic properties of the core, and the cross-section and material of the secondary winding are required. The geometry of the transformer core is composed of two straight parts (154 mm long) and two semicircles of 92 mm inner and 105 mm outer radius. Figure 3a shows the geometry and Figure 3b the cross-section of the magnetic core.

**Figure 4:**
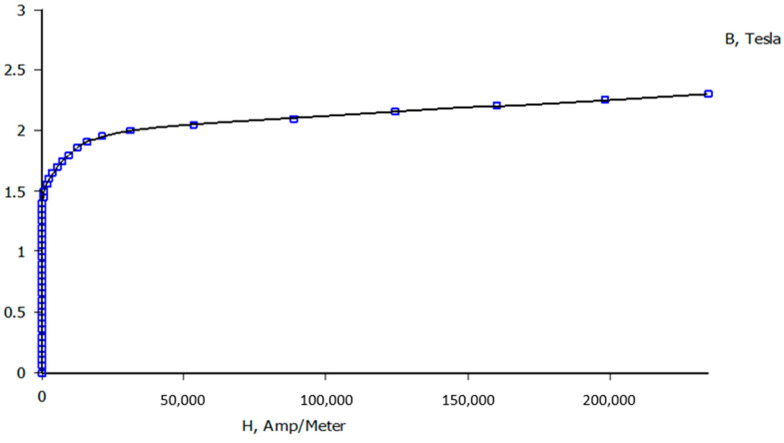
Magnetization curve of M-15 steel.

Regarding the magnetic core properties, a material from the FEMM library (M-15 Steel) was selected. This material closely matches the known properties of the actual SiFe core (its knee point corresponds to a secondary voltage of 26 V). Figure 4 shows the B-H curve of the M-15 steel [26].

The secondary winding is composed of 500 turns of a copper conductor with 0.8 mm diameter. The primary winding is the conductor that passes through the transformer. It is modeled as a circle of 9 mm radius made of copper.

## 3. Results

This section presents the experimental and the finite elements simulations results of the two specifically manufactured non-toroidal shape primary pass-through current transformers, as described in Section 2.

### 3.1. Experimental Laboratory Tests

The tests for protection current transformers should be performed according to the standard IEC 61869-2. The standard allows two possibilities, indirect and direct tests. The direct tests are more complex as it is necessary to inject several times the primary rated current and in some cases this means thousands of amperes. On the other hand, the indirect tests only request a low-power voltage supply. The indirect method is only suitable for low leakage reactance current transformers. Despite these tests having been performed by the manufacturer, they were repeated in the laboratory for cross checking the results.

#### 3.1.1. Indirect Method

The indirect method requires mainly two tests, secondary winding resistance measurement and the excitation test.

The secondary resistances have been measured in the laboratory with a 4-wires ohmmeter at 20 °C. The obtained results are 1.15 Ω and 1.14 Ω for the two tested transformers, distributed and partially distributed secondaries, respectively.

**Figure 5:**
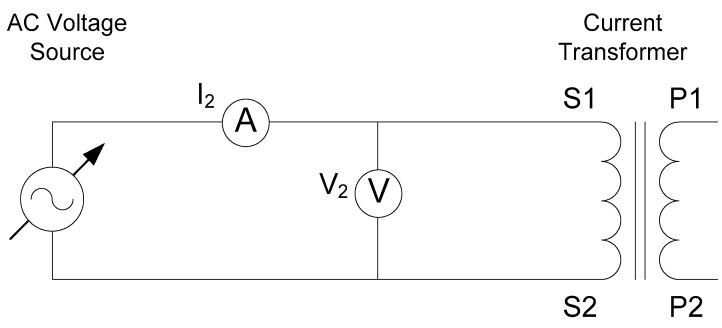
Excitation test layout.

The excitation test represents the iron core saturation. This test is performed by applying a sinusoidal voltage (V_2_) in the secondary winding while the primary winding is opened, as shown in the Figure 5. The injected voltage produces a magnetic flux in the core, and the value of the rms current should be recorded. The saturation characteristic of the iron core is determined when an increase of 10% of the voltage represents an increase of 50% in the excitation current.

**Figure 6:**
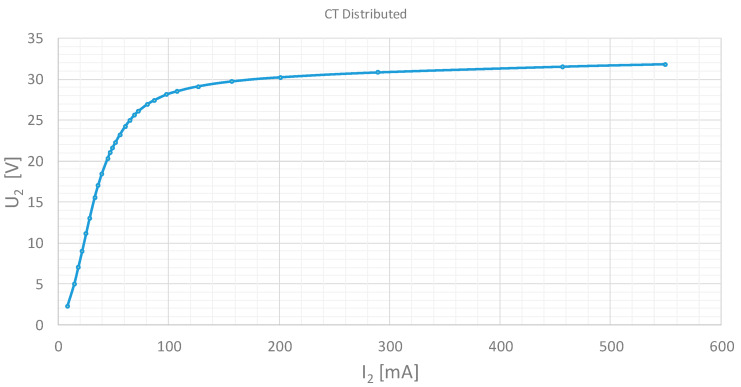
Excitation test result. Current transformer 500/1A 5P10 1VA. Distributed secondary winding.

**Figure 7:**
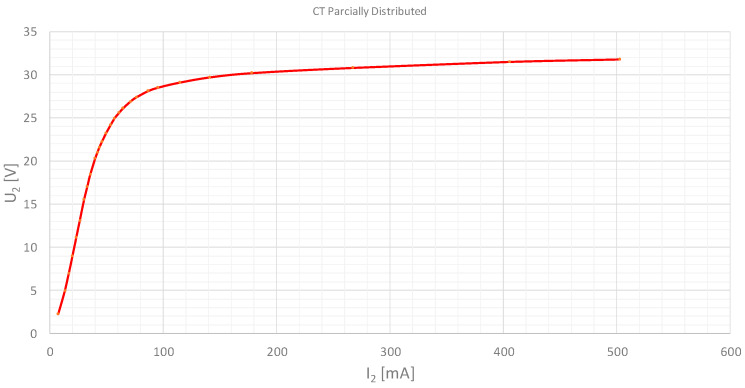
Excitation test result. Current transformer 500/1A 5P10 1VA. Partially distributed secondary winding.

The applied voltage (V_2_) is represented versus the magnetization current (I_2_). The test results are shown in Figure 6 and Figure 7 for the distributed and partially distributed secondary transformers respectively. These tests have been performed in our laboratory and the tables have been made by the authors.

The protection current transformers should operate with a certain limited error at several times the primary rated current. In the case of these transformers, the accuracy class is 5P10, so the maximum allowable composite error is 5% at 10 times the rated primary current. The induced voltage in the secondary winding under this operating condition is known as the electromotive force at the accuracy limit current (*E_ALF_*). It is calculated according to Equation (1). In other words, this is the required electromotive force in the secondary winding when a current *ALF* times the rated current flows in the primary winding.

(1) $$E_{ALF}=ALF\cdot I_{2N}\cdot \sqrt{{\left(R_{\;CT}+R_{b}\right)}^{2}+X_{b}{}^{2}}$$

where *E_ALF_*—Electromotive force required at the accuracy limit current (knee point); *ALF*—Accuracy limit factor; *I_2N_*—Secondary rated current; *R_CT_*—Secondary winding resistance; *R_b_*—Burden resistance; *X_b_*—Burden reactance.

As explained before, the *ALF* is 10. The burden load is 1 Ω and is considered purely resistive. The secondary measured resistances were 1.15 Ω and 1.14 Ω.

From the excitation tests, according to the standard, the excitation current *I_K_* at the excitation voltage *E_ALF_* is determined. This current (*I_K_*) divided by the product of the rated secondary current multiplied by the accuracy limit factor must not exceed the limit for the composite error. This relation corresponds to Equation (2).

(2) $$\varepsilon_{C}=\frac{I_{K}}{AFL\;I_{2N}}100\;\left[\%\right]$$

*ε_c_*—Composite Error; *I_K_*—Excitation current at the knee point.

A summary of the results of the indirect method is shown in Table 2. According to IEC 61869-2 [23], as the obtained composite errors are below 5%, both transformers pass the indirect test.

#### 3.1.2. Direct Method

The direct method is based on the injection of a high current in the primary winding, while the rated burden is connected to the secondary terminals. The injected current is increased from the rated current to several times the rated current and up to the accuracy limit factor. The secondary current should be measured and compared to the theoretical secondary current, calculated with the current ratio. The composite error is calculated as Equation (3).

(3) $$\varepsilon_{c}=\frac{\sqrt{\frac{1}{T}\cdot \int_{0}^{T}{\left(R_{t}\cdot i_{2}-i_{1}\right)}^{2}dt}}{I_{1}}$$

where *ε_c_*—Composite Error; *R_t_*—Transformer current ratio; *I_1_*—Primary current (RMS); *i_1_*, *i_2_*—Instantaneous primary and secondary currents respectively; *T*—Period.

**Figure 8:**
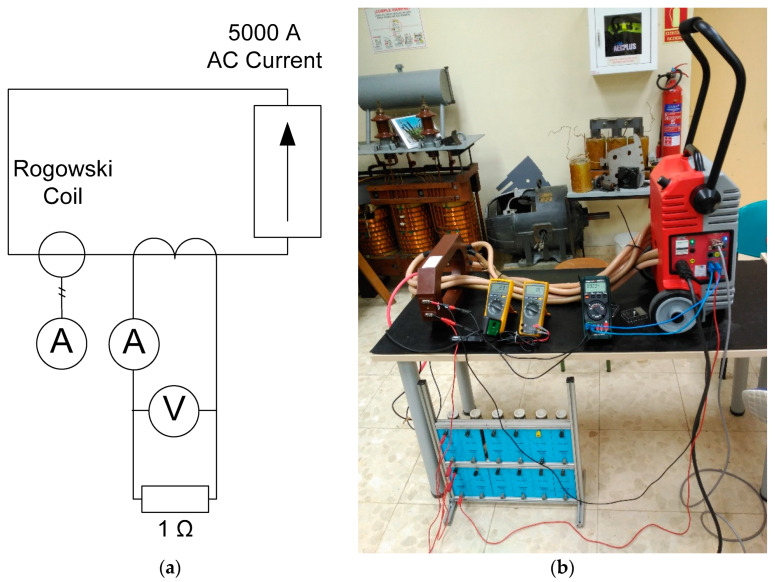
Experimental setup. (**a**) Simplified diagram. (**b**) Laboratory injection test setup.

The experimental simplified diagram is shown in Figure 8a. The setup is composed of a high current injection test equipment up to 5000 A, a Rogowski coil for direct measurement of the primary current, the current transformer under test, and a resistance connected to the secondary. An additional ammeter and a voltmeter are needed for the secondary.

The experimental laboratory setup is displayed in Figure 8b.

Both current transformers are tested from the rated current (500 A) to 10 times the rated current (5000 A). The injection test equipment and the Rogowski coil measure the primary current. The secondary winding of the current transformers has a 1 Ω resistor connected to them. Finally, an ammeter measures the secondary current.

The results of the primary injection tests are presented in Table 3.

### 3.2. Finite Element Simulations

The finite element simulations have been performed with the FEMM (Finite Element Method Magnetics) software. The simulated model has been developed based on the geometrical features presented in Figure 4. Magnetic steel M-15 has been chosen among the various magnetic materials available in the software’s library to represent the iron core. The secondary turns are 500, while the primary current is simulated by a cable in the center of the transformer. Several simulations, corresponding to the previously described experimental tests, were performed.

**Figure 9:**
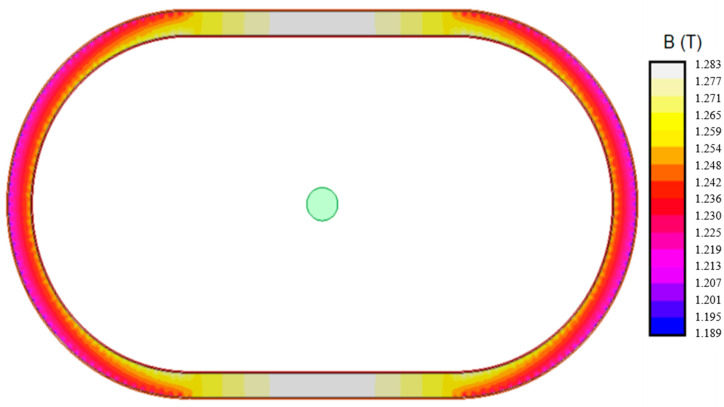
Flux density. Current transformer 500/1A 5P10 1VA. Distributed secondary winding. Primary current 5002 A, secondary current 9.719 A.

Figure 9 shows the results of a simulation where the accuracy limit current (5002 A) is injected in the primary pass-through winding, while 9.719 A are injected in the secondary winding. It can be clearly seen than the flux density is not uniform around the iron core, but the distribution is doubly symmetrical with respect to the vertical and horizontal axis. The areas with a higher flux density are the straight part of the iron core, especially in the part where the primary is closer. On the other hand, the flux density in the round parts of the iron core are slightly lower.

**Figure 10:**
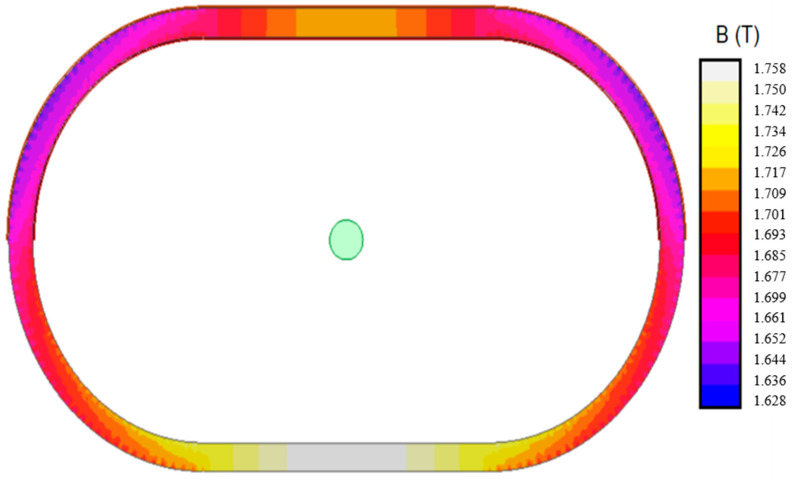
Flux density. Current transformer 500/1A 5P10 1VA. Partially distributed secondary winding. Primary current 5002 A, secondary current 6.021 A.

Figure 10 shows a similar case but in the current transformer with its secondary winding partially distributed. In this case, the accuracy limit current (5002 A) is injected in the primary pass-through winding and 6.021 A in the secondary winding.

In this case (Figure 10), the flux density is different in the upper and lower part of the iron core. The flux density is symmetrical in this case, but only with respect to a vertical axis. The secondary winding is placed only in the upper part of the iron core. It can be clearly observed than in this area the flux density has lower values. In the bottom part of the iron core, where there is no secondary winding, the flux density has greater values.

## 4. Discussion

The performance of two similar protection current transformers have been evaluated. One of the most important features of this transformer type is that it should not saturate at the accuracy limit factor current. If they do saturate, their measurements are not deemed reliable. As a consequence, the protection systems, the reliability of which is highly dependent on these measurements, may malfunction. There are two types of malfunctions, false positive (tripping by an inexistent fault) and false negative (no tripping with an actual fault). Therefore, several damages such as destruction of equipment (false negatives) and blackouts (false positives) can happen due to erroneous readings of the current transformers.

From this paper’s measurements, a bad performance of the partially distributed secondary transformer is clearly observed. This transformer should have an error lower than 5% along its entire measurement range. However, for currents larger than 1500 A, its error is larger. For its maximum current (5000 A), its error is circa 40%. Regarding the uniformly distributed secondary current transformer, its maximum composite error (3.12% at 3027 A) is lower than the maximum allowed error (5%).

In order to better understand the significance of these composite errors, an assessment of them is needed. To this aim, it should be taken into account the errors inherent to the instrumentation used. In this case, the Rogowski coil has a measurement error lower than 1% and the ammeters have error lower than ≤0.5% + five digits. As the value of the burden resistor used in the tests for both transformers is the same, its tolerance does not affect the comparison of both transformers. Therefore, assuming that the Ammeters and Rogowski coil errors are independent, the error in the computation of the composite error is:

$$∆\varepsilon_{c}=\sqrt{{\left(\frac{R_{t}\cdot ∆I_{2}}{I_{1}}\right)}^{2}+{\left(\frac{∆I_{1}}{I_{1}}\right)}^{2}}=\sqrt{2\cdot {\left(0.5\right)}^{2}+{\left(1\right)}^{2}}≅1,2\%$$

Consequently, it is clear that the non-distributed secondary winding current transformer has composite errors much larger than their accuracy class limit. On the contrary, the distributed secondary winding current transformer has composite errors lower than their accuracy class limit.

Both transformers were simulated by finite elements under the maximum current operating condition. These simulations show the different magnetic performance of both transformers: The distributed secondary transformer has a maximum flux density of 1.28 T at the parts of the core nearest to the primary conductor. These parts correspond to the midpoint of the straight segment of the iron core. The minimum flux density is 1.20 T at the parts of the core farthest to the primary conductor. These parts are placed in the center of the curved parts of the iron core. This transformer has symmetry along vertical and horizontal axis, so all results exhibit the same symmetry. Every flux density value is below the knee point, so, this core is not saturated.

On the other hand, the partially distributed secondary winding transformer reaches a maximum flux density of 1.75 T in the magnetic core nearest to the primary conductor without secondary winding, i.e., the bottom part of the iron core. In the part closest to the primary conductor, a flux density of 1.7 T is reached. This transformer has symmetry only along the vertical axis, so all results exhibit this symmetry. It should be pointed out that, according to Figure 4, every flux density is over the knee point. Therefore, this core is saturated.

The results obtained in the simulations explain the differences in the performance of both transformers. As the partially distributed secondary winding transformer core is saturated, the relation between their secondary and primary currents is no longer linear. Therefore, their measurements are not reliable. On the contrary, as the distributed secondary winding transformer is not saturated, the relation between their secondary and primary currents is linear. Therefore, their measurements are reliable.

This happens because primary current magnetizes the iron core and secondary current demagnetizes it. The part of the core without secondary winding has no demagnetizing current, so it saturates. Due to this saturation, the actual secondary current is lower, which reduces the demagnetization effect, increasing saturation along the whole magnetic core.

## 5. Conclusions

The use of non-toroidal shape pass-through current transformers is increasing in high current machines with several cable per phase, thanks to its easy installation. Nowadays, the compact SF_6_ gas insulated switchgear is very common where the current transformers are pass-through type and are installed outside the panels. In this type of installation, the use of non-toroidal shape pass-through current transformers is rising.

This paper presents two similar non-toroidal shape pass-through protection current transformers with two different secondary windings distributions. In one of the transformers, the secondary winding has been distributed uniformly around the iron core. The other transformer has the secondary distributed only along half of the iron core.

The indirect test method is very simple, but it is not suitable for this type of transformer. Although both current transformers have passed the indirect tests, the first one can correctly operate up to its accuracy current limit (10 *I_N_*) but not the second one.

The finite element simulations reveal the asymmetry in the flux density distribution in non-toroidal shape current transformers. The straight part of the core has a greater flux density. This should be taken into consideration for the design, even in case of distributed secondary windings.

Another important conclusion is than the non-uniform distribution of the secondary winding produces an additional asymmetry in the flux density distribution. So, the iron core can reach the saturation level easier and consequently lead to a malfunction of the current transformer.

The standard IEC-61869 specifies that the indirect test is valid only for low leakage reactance current transformers. Among the conditions to consider that a current transformer is of low leakage reactance, its iron core should be substantially toroidal. However, the uniformly distributed secondary winding current transformer performs correctly in both the indirect and direct tests. As shown by the finite element simulations, the flux density is below the knee point. So, there is no saturation in the magnetic core. For this reason, this transformer performs correctly.

Besides, the difference between maximum and minimum flux density along the magnetic core depends on its relative dimensions. So, as the toroidal were more elongated, this difference is stronger for a single conductor. As a consequence, the current distribution inside the transformer window should be carefully analyzed.

Regarding partially distributed secondary current transformers, they do not pass the direct test despite their correct performance under indirect tests. So, these transformers cannot be accurately checked using only indirect tests.

The main conclusion of this paper is that protection current transformers should be tested with primary injection, especially when there are doubts about whether they classify as low leakage types.

Another important conclusion is that the non-toroidal shape pass through current transformers cannot be considered as low leakage type according to the standard. Therefore, they should be tested using the direct method test.

A malfunction of the protection current transformers can cause severe damages such as destruction of equipment (false negatives) and blackouts (false positives). Therefore, their correct performance is of paramount importance to power and industrial systems.

## Figures and Tables

**Table 1:** Current transformers characteristics.

Variable	Value
Rated primary current	500 A
Rated secondary current	1 A
Rated burden power	1 VA
Rated burden load	1 Ω
Accuracy class	5P10
Accuracy limit factor	10
Frequency	50 Hz
Rated voltage	0.72/3 kV
Short-time thermal current	50 kA; 1 s

**Table 2:** Current transformers indirect method test results.

	Parameter	CT Distributed	CT Partially Distributed
R_CT_	Secondary resistance	1.15 Ω	1.14 Ω
E_ALF_	Electromotive force required at the accuracy limit current	21.5 V	21.4 V
I_K_	Excitation current at the knee point	49.1 mA	43.7 mA
ε_c_	Composite error	0.49%	0.43%

**Table 3:** Current transformers direct method experimental test results.

	CT Distributed		CT Partially Distributed	
Primary Current [A]	Secondary Current [A]	Composite Error [%]	Secondary Current [A]	Composite Error [%]
509	1.021	2.16	1.005	6.60
1020	2.042	2.51	1.999	3.95
1516	3.028	2.77	2.873	7.75
2017	4.024	2.96	3.369	17.72
2520	5.012	3.08	3.887	24.08
3027	6.009	3.12	4.362	29.12
3535	6.988	3.09	4.811	33.07
3995	7.809	2.99	5.218	35.51
4486	8.742	2.82	5.598	38.64
5002	9.719	2.57	6.021	40.93
